# Outcomes and vaccination patterns against COVID-19 in a cohort of sickle cell disease patients in the state of Rio de Janeiro

**DOI:** 10.1016/j.htct.2025.103824

**Published:** 2025-04-10

**Authors:** Claudia de Alvarenga Maximo, Jorge Francisco da Cunha Pinto, Fabiana Canedo Pinto, Patrícia Brasil

**Affiliations:** a Instituto Estadual de Hematologia Arthur de Siqueira Cavalcanti – HEMORIO, Rio de Janeiro, State of Rio de Janeiro, Brazil; b Universidade Federal do Estado do Rio de Janeiro – UNIRIO, Rio de Janeiro, State of Rio de Janeiro, Brazil; c Instituto de Pesquisa Clínica Evandro Chagas – FIOCRUZ, Rio de Janeiro, State of Rio de Janeiro, Brazil

**Keywords:** Covid-19 vaccine, Sickle cell disease, Vaccination awareness

## Abstract

**Background:**

Patients with sickle cell disease were presumed to be at high risk for severe COVID-19 outcomes due to their compromised immunity and chronic comorbidities. This study aimed to evaluate vaccination patterns, healthcare utilization, and clinical outcomes in a cohort of sickle cell disease patients during the COVID-19 pandemic in Rio de Janeiro.

**Methods:**

A total of 289 over 18-year-old patients from the Epidemiology and Donor Evaluation Study (REDS-III) Brazil sickle cell disease cohort were followed between January 2021 and August 2023. Sociodemographic data, emergency department visits, hospitalizations, mortality rates, and COVID-19 vaccination status were collected. SARS-CoV-2 infection was confirmed by reverse transcription polymerase chain reaction testing for symptomatic or hospitalized patients.

**Results:**

Of the participants, 89.2% completed the primary vaccination schedule, 62.2% received the first booster, 30% the second booster, and 4.1% completed all five doses. Emergency visits increased slightly during the pandemic but were primarily due to vaso-occlusive crises. Of the 119 patients tested for SARS-CoV-2, six were positive, presenting mild symptoms with no COVID-19-related deaths. Vaccination rates in the cohort were similar to those in the general population, with Oxford/AstraZeneca and Pfizer being the most used vaccines.

**Discussion:**

The findings suggest that COVID-19 infection was not a significant trigger for vaso-occlusive crises or severe disease outcomes. High vaccination adherence likely played a key role in preventing severe COVID-19, alongside other factors such as social isolation and herd immunity. However, the overlap between symptoms of vaso-occlusive crises and COVID-19 may have caused diagnostic challenges. Importantly, the low morbidity and mortality observed emphasize the protective effect of vaccines, despite the presence of thromboplastic activity and pro-inflammatory states inherent to sickle cell disease. Addressing vaccine hesitancy remains crucial, particularly as booster doses show declining adherence.

**Conclusion:**

COVID-19 had a limited clinical impact on this cohort, with no significant role in triggering vaso-occlusive crises or severe outcomes. High vaccination rates and potential environmental or biological factors may have contributed to this protective effect.

## Introduction

COVID-19 emerged in China at the end of 2019, and due to the high transmissibility of the SARS-CoV-2 virus through respiratory droplets, it was responsible for nearly 800 million cases worldwide by February 2024.^1^ In Brazil, there were approximately 38.5 million cases with over 709,000 deaths in the same period.^2^ Initially, the only strategy for governments to mitigate transmission was social isolation, however by the end of the first pandemic year, the first vaccines were made available by the pharmaceutical industry, significantly reducing morbidity and mortality. Vaccination began in January 2021, with the first doses of COVID-19-Coronavac-Sinovac/Butantan (Coronavac) being administered, prioritizing the most vulnerable groups, such as the elderly and those with chronic diseases, including sickle cell disease (SCD). This hereditary hemoglobinopathy, characterized by multiple comorbidities, posed a high presumed risk of complications if patients contracted SARS-CoV-2. The polymerization of red blood cells containing hemoglobin (Hb) S leads to the main pathophysiological mechanisms of the disease, namely vaso-occlusion and hemolysis. These events cause vascular and endothelial dysfunction through inflammatory and pro-thrombotic mechanisms resulting in acute events and chronic organ damage.^3^ Pain crises due to vaso-occlusion are the hallmark of SCD with the main pathophysiological mechanisms being: 1) polymerization of deoxyhemoglobin S, 2) Sickling of red blood cells, 3) microvascular occlusion by sickled cells, 4) tissue ischemia, 5) tissue damage due to hypoxia and 6) stimulation of peripheral nerve endings that lead to pain perception.^4^

The lungs are frequently affected, with acute chest syndrome (ACS) being a severe complication and the leading cause of death. Patients have compromised immunity due to hyposplenism, making them susceptible to infectious diseases, sepsis, chronic kidney disease, and pulmonary hypertension, justifying their inclusion in the priority group.^4^

COVID-19 primarily affects the respiratory system with the release of inflammatory cytokines, presenting symptoms ranging from mild to severe, potentially leading to circulatory and thromboembolic complications, and death from pulmonary involvement and multiple organ failure. By January 2021, Brazil began offering vaccines, and by 2023, monovalent vaccines such as Coronavac, COVID-19-RNAm Pfizer-Comirnaty (Pfizer), Covishield-Oxford/Fiocruz (AstraZeneca), Janssen (Janssen), and the COVID-19-RNAm Pfizer (Comirnaty) bivalent vaccine (Bivalent), introduced after the emergence of the Omicron variant, were available. The primary vaccination schedule consists of two doses, followed by a first booster, a second booster, and a third booster including the bivalent vaccine.^5^

The objective of this study was to investigate the vaccination pattern and its association with clinic outcomes and healthcare utilization of a cohort of SCD patients at the blood center of Rio de Janeiro in the pandemic period. Given that patients with SCD are at high risk for COVID-19 complications, it was hypothesized that this population may have experienced worse clinical outcomes and increased utilization of healthcare services due to COVID-19 infection.

## Methods

### Study population and selection criteria

All over 18-year-old patients, who were participating in the multicenter cohort study, Epidemiology and Donor Evaluation Study (REDS-III) Brazil Sickle Cell Disease, of the blood center of Rio de Janeiro were selected. REDS-III is a longitudinal cohort project involving six SCD treatment centers across Brazil in collaboration with researchers from the Blood Systems Research Institute in California (USA) since 2013.^6^ Out of the total patients of Hemorio, 721 were selected by REDS-III based using sample size calculations. Of these, 337 met the eligibility criteria for this study. A consent form was signed by 289 patients.

### Variables of interest

Sociodemographic data, including SCD genotype, age, race and educational attainment, were collected from medical records as part of the REDS database. Additionally, information on numbers of emergency visits and hospitalizations was gathered for three time periods to compare the pre-pandemic period (2018–2019) to the pandemic (2020–April 2022) and to the post-pandemic period, after the end of the international public health emergency (May 2022–2023). The reasons for emergency visits in the periods were described.

Only patients presenting with respiratory symptoms and those hospitalized for any reason were tested for SARS-CoV-2 using reverse transcription polymerase chain reaction (RT-PCR) as part of standard care.

Mortality rates were calculated based on the number of deaths within the cohort during the study period, and deaths were analyzed in terms of their dates and causes.

The COVID-19 vaccination status of participants was obtained from the National Immunization Program (NIP) website from January 2021 to August 2023. For this study, the vaccines offered in sequence were categorized as the first dose, second dose, first booster, second booster, and bivalent. Participants who received all five doses available from NIP were considered to have completed the vaccination schedule. The sociodemographic profiles of the vaccinated and unvaccinated populations were assessed. A comparison of the vaccination rate of the cohort participants with the population of the municipality of Rio de Janeiro was made.

This study was approved by the Hemorio Ethics Committee under register number 6089,121 and conducted according to the revised 2008 Helsinki Declaration.

### Statistical analysis

Sociodemographic and clinical variables are described using descriptive statistics, including means, standard deviations, and absolute and relative frequencies.

For each of the two periods, the incidence rate of emergency room visits was calculated as the number of visits per person-months at risk. The incidence rates between periods were compared using Poisson regression.^7^ In the Poisson model, the dependent variable was the number of emergency visits, and the independent variable was a binary variable indicating whether the visit was during the pre-pandemic or pandemic period. The incidence rate ratio and its 95% confidence interval were estimated using the GLM package in R 4.3.1 8.^8^ A ratio greater than one with a confidence interval that does not overlap with one would represent a significantly greater risk of emergency visits during the pandemic, whereas a confidence interval overlapping one would indicate no significant association between the pandemic and emergency visits.

## Results

Of the 289 participants, 116 (40%) were male, and 173 (60%) were female, with a mean age of 34.3 ± 13.2 years (range: 18–72 years). About one-half (*n* = 147; 51%) were identified as mixed race, 114 (39%) as Black, 25 (9%) as White, and three (1%) as Asian. Regarding genotype, 227 (78.5%) were Hb SS, 42 (14.5%) Hb SC, 12 (4.1%) Hb SB^0^, seven (2.4%) Hb SB^+^, and one Hb SD. Educational attainment varied with 87 (30.1%) individuals having basic education, 50 (17.3%) with completed basic education, 103 (35.6%) with secondary education, 19 (6.6%) had completed technical courses, 27 (9.3%) had higher education, one participant with a master's degree, and two were illiterate.

**Figure 1:**
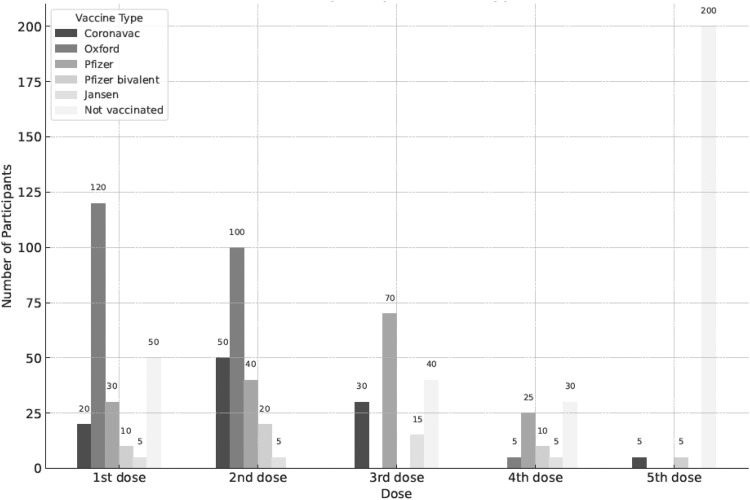
Number of participants per vaccine type and dose.

Most participants (89.2%) completed the primary COVID-19 vaccination schedule with two doses. Of these, 62.2% received the first booster dose (third dose), while 30% completed up to the second booster dose (fourth dose). Only 4.1% received all five recommended doses according to the Brazilian vaccination schedule, and 11.7% of participants received the bivalent vaccine between the fourth and fifth doses. Five males and four females with varied educational backgrounds did not get vaccinated. The distribution of vaccine doses by sex, education level, and age group is shown in Table 1. There was no significant association between education level, age group, and sex regarding vaccination, except for the second booster dose, where more women were vaccinated. The most used vaccine from the first to the fourth dose was the Oxford/AstraZeneca, followed by Coronavac. The distribution of vaccine types by dose is shown in Figure 1.

**Table 1:** Distribution of vaccine doses by sex, educational attainment, and age group.

Variable	1st Dose n (%)	2nd Dose n (%)	1st Booster n (%)	2nd Booster n (%)	Bivalent n (%)	None n (%)	All n (%)
**Sex**							
Male	111 (39.6)	99 (35.3)	67 (23.9)	29 (10.4)	5 (1.8)	5 (2.9)	5 (4.3)
Female	168 (60.0)	161 (57.5)	115 (41.1)	58 (20.7)	7 (2.5)	4 (3.4)	7 (4.0)
**Education**							
Illiterate	2 (100)	2 (100)	1 (50)	–	–	–	–
Primary school	83 (95.4)	76 (87.3)	53 (60.9)	26 (29.9)	3 (3.4)	5 (5.7)	3 (3.4)
Secondary school	48 (97.9)	42 (85.7)	29 (59.1)	9 (18.3)	3 (6.1)	1 (2.0)	2 (4.0)
High school	101 (98.0)	98 (95.1)	68 (66.0)	39 (37.8)	3 (2.9)	2 (1.9)	5 (4.8)
Adult education	1 (100)	1 (100)	1 (100)	–	–	–	–
Technical school	17 (89.4)	13 (68.4)	10 (52.6)	2 (10.5)	–	1 (5.2)	–
Higher education	26 (96.2)	26 (96.2)	17 (62.9)	8 (29.6)	3 (11.1)	–	2 (7.4)
Postgraduation	1 (100)	1 (100)	1 (100)	1 (100)	–	–	–
**Age group**							
18–29	133 (98.5)	122 (90.4)	74 (54.8)	25 (18.5)	5 (3.7)	1 (0.7)	5 (3.7)
30–39	62 (95.4)	58 (89.2)	45 (69.2)	26 (40.0)	3 (4.6)	3 (4.6)	3 (4.6)
40–49	39 (90.6)	36 (83.7)	26 (60.5)	12 (27.9)	1 (2.3)	4 (9.3)	1 (2.3)
50–59	26 (92.8)	27 (96.4)	20 (71.4)	12 (42.8)	1 (3.6)	1 (3.6)	1 (3.6)
60+	18 (100)	17 (94.4)	17 (94.4)	12 (66.7)	2 (11.1)	–	2 (11.1)

In the pre-pandemic period, 77 patients made a total of 1020 emergency room visits. During the pandemic, 88 patients made 1416 visits, while in the post-pandemic period, 92 patients accounted for 1286 visits (Table 2). Most individuals seen in the pre-pandemic and pandemic periods were the same, with SCD-related pain crises and the need for blood transfusions being the leading reasons for emergency department visits.

**Table 2:** Emergency room visits.

Period	Number of patients	Number of visits
Pre-pandemic	77	1020
Pandemic	88	1416
Post-pandemic	92	1286

Before the pandemic, the incidence rate of emergency visits was 0.23 visits per person-month at risk. During the pandemic, the rate was 0.31 visits/person-month. The incidence rate ratio of the Poisson model was 0.99 (95% confidence interval: 0.93–1.07, p-value = 0.92).

During the study, 119 patients underwent RT-PCR testing for SARS-CoV-2. Of these, 52 were unvaccinated and 67 were vaccinated. Among the unvaccinated, four tested positive. They were hospitalized for vaso-occlusive crises (VOCr) but only one had respiratory symptoms and anosmia. They were discharged in three to five days. Among the vaccinated, two tested positive. They were also admitted due to VOCr and discharged after pain control.

There were two deaths during the pandemic, configuring a mortality rate of 0.7%: a 34-year-old man died due to liver failure on October 10, 2022. He tested positive for SARS-CoV-2 by RT-PCR before being vaccinated on April 20, 2021 and received two doses of the Coronavac vaccine on August 16 and September 23, 2021. The second was a 19-year-old woman who died due to ACS and septic shock on October 8, 2022. When she was admitted, she exhibited fatigue, fever, headache, and chest pain. No Testing for COVID-19 was done at this time. She had mild COVID-19 with a positive RT-PCR test 18 months before and received two doses of the Oxford/AstraZeneca vaccine on June 28 and October 4, 2021, one year before her death.

**Figure 2:**
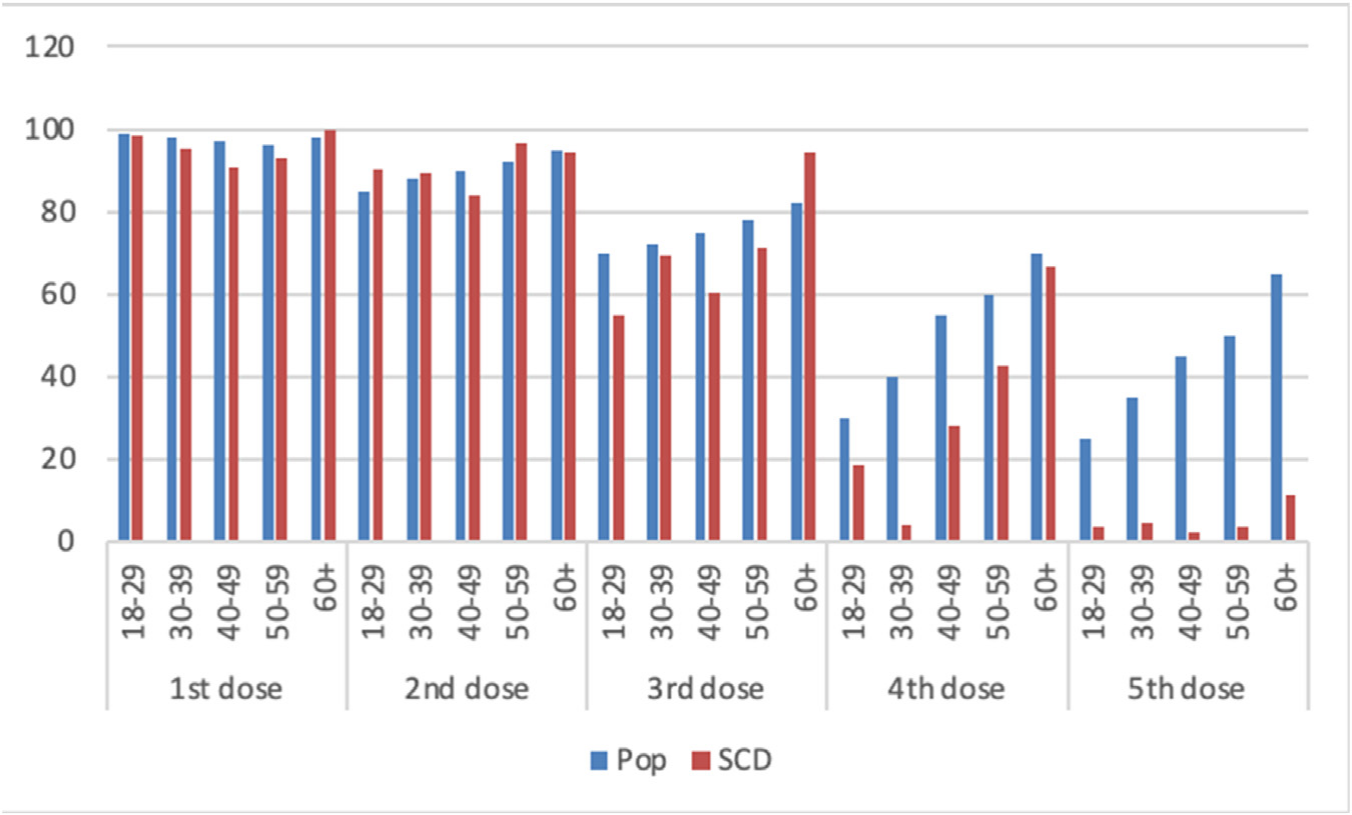
Comparison of vaccination by age group between the sickle cell disease (SCD) cohort and the population of the city of Rio de Janeiro (Pop).

The SCD cohort had similar vaccination rates to the general population of Rio de Janeiro as seen in Figure 2, except for the fourth (p-value <0.01) and fifth (p-value <0.01) doses, which were lower. Up to the primary vaccination scheme of two doses, the vaccination pattern was the same for all age groups. From the first booster onwards, there was a reduction in the vaccination rate in the SCD population. The second booster was not considered due to insufficient time for evaluation.

## Discussion

Despite the high reliance of SCD patients on emergency healthcare, particularly for VOCr, the number of emergency visits did not increase significantly during the COVID-19 pandemic. This may be attributed to robust antibody responses post-vaccination, as observed in previous studies.^9^ SCD is associated with increased thromboplastic activity, characterized by heightened platelet activation, elevated markers of thrombin generation, and increased tissue factor expression, along with reduced levels of natural anticoagulants, even in the basal state and in the absence of acute events such as VOCr. However, it remains unclear whether this thromboplastic activity contributes to the pathophysiology of VOCr or, as is more widely accepted, merely reflects an acute-phase response to these events.^10^^,^^11^ Notably, SARS-CoV-2 infection has been identified as a potent inducer of thrombosis, raising additional concerns in this already vulnerable population.^11^ Despite these concerns, this study did not identify an increase in VOCr following COVID-19 vaccination, alleviating fears that vaccines might trigger vaso-occlusive events.^9^ The possibility of VOCr triggered by mild or asymptomatic SARS-CoV-2 infection in patients with SCD cannot be entirely ruled out. This is a limitation of the current study, as most patients who presented to the emergency department with pain were not tested for COVID-19. One hundred and nineteen patients underwent PT-PCR testing for SARS-CoV-2 due to emergency admissions, as cohort wards were separated for patients with positive tests at that time. Only six tested positive (5%). All presented with VOCr but only one had respiratory symptoms related to COVID-19. These data are quite similar to those published by Konte et al., who performed RT-PCR tests for SARS-CoV-2 on 104 admissions for VOCr of 62 patients with SCD. Only five tested positive (4.8%), and only one had associated respiratory symptoms.^12^ Thus, as discussed above, it is not possible to state whether the VOCr were triggered by COVID-19 or not. However, the low incidence of positive tests suggests that COVID-19 could have been at most an asymptomatic co-incidental infection.

The Brazilian National Immunization Program is a global reference, providing extensive free immunobiologicals to the population. SCD patients were included in the National Operational Plan for COVID-19 vaccination due to their compromised immunity resulting from functional hyposplenism and systemic vasculopathy.^5^

Vaccination adherence of individuals with SCD was comparable to the general population in Rio de Janeiro for the primary two-dose schedule. However, lower rates were observed for the fourth and fifth doses, likely due to the study period ending shortly after the rollout of bivalent vaccine in February 2023.^13^ The most frequently used vaccine in the present cohort was the Oxford/AstraZeneca for the first two doses, followed by the Pfizer vaccine starting from the third dose. These were also the most used vaccines in Brazil in 2021.^14^

A study on vaccination intention assessment indicated that people with SCD, similar to other groups with chronic diseases, were less willing to get vaccinated, despite being vulnerable.^15^^,^^16^ In contrast to studies on vaccine hesitancy, this study identified high adherence to the primary vaccination schedule, with a slight reduction for the first booster dose among younger individuals. Encouragement by healthcare professionals for individuals to get vaccinated, the widespread availability of vaccines to priority groups, and the fear of contracting and dying from COVID-19 were likely more influential than concerns over potential vaccine side effects.

The absence of vaccination in nine individuals who did not receive any vaccine dose might be explained by a combination of factors: fear of side effects, concerns over triggering VOCr and thrombosis, and, most notably, a disbelief in vaccines. These factors have been highlighted in other studies, such as one conducted in a treatment center in Saudi Arabia with 147 SCD patients, which showed that only 35% had been vaccinated, revealing high vaccine hesitancy. The main reason given by the lack of vaccination was the fear of developing brain clots and other side effects.^17^^,^^18^ Despite not being vaccinated, only one patient who tested positive by RT-PCR exhibited mild symptoms of COVID-19 in September 2022, without requiring hospitalization. The other patients, despite frequently using the healthcare system, either did not contract the disease or it went unnoticed, due to the presence of non-specific symptoms, since some clinical manifestations of COVID-19 are also commonly seen in SCD.^4^ Social isolation and herd immunity could explain the lack of illness or severe COVID-19 in this group.

COVID-19 vaccines have been shown to be safe and do not cause significant or more severe adverse effects in people with SCD, as evidenced by Han et al. Their work also observed that the patients who completed the primary vaccination regimen had a 70% reduction in the risk of SARS-CoV-2 infection before the emergence of the Omicron variant.^19^ In this study, 89% patients were fully vaccinated and one died of ACS that could not be dissociated from SARS-CoV-2 infection. No association was found between vaccination and sex, education, and age, possibly due to the sample size. Also, the adverse effects of the vaccines in this population were not evaluated. Nonetheless the adverse effects of COVID-19 vaccines are mostly mild, such as local pain, fever, fatigue, headache, muscle pain, chills, and diarrhea.^20^ These symptoms are also commonly seen in SCD and often lead patients to seek emergency care.

Varelas et al.^21^ studied the immune response after vaccination against Sars-Cov-2 in a group of SCD patients regarding the production of neutralizing antibodies (nAbs) and the increase in C5b-9 levels: nAbs >50% are highly protective. The study showed a satisfactory immune response, especially after the second dose, with 50% of the patients studied achieving nAb levels ≥50% and a significant increase in C5b-9 above baseline levels. As in the present sample, 89% completed the primary vaccination schedule: this may have been the reason for the low morbidity from COVID-19 and the low mortality rate of 0.7% (not related to COVID-19) in this group, when compared to the general population in Rio de Janeiro during the years 2020 (12.6%), 2021 (14.3%), 2022 (11.8%), and 2023 (11.2%).^22^

Both patients who died had received two doses of the COVID-19 vaccine approximately one year before their deaths. They presented with severe SCD complications, including liver failure and ACS with septic shock. While SARS-CoV-2 infection cannot be entirely ruled out – particularly given the limitations of RT-PCR testing – neither patient had been vaccinated against the Omicron variant. This underscores the potential role of waning immunity and the importance of timely booster doses, especially for vulnerable populations, such as individuals with SCD. Guo et al.^24^ studied a cohort of patients who had recovered from COVID-19 to investigate the durability and cross-reactivity of immunological memory acquired from natural infection against SARS-CoV-2. They found that neutralizing antibodies continually declined but SARS-CoV-2-specific memory B-cell and T-cell responses were maintained for at least two years and the recall immune responses could limit viral replication and reduce disease severity after re-infection. Furthermore, humoral immunity was boosted against the prototype and Omicron sublineages in individuals who were infected by the prototype and who subsequently received the inactivated vaccine.^23^^,^^24^ This was the case for the two patients who died: after having had COVID-19 they died about a year after being vaccinated.

It is true that the Omicron variant and its sublineages have posed an additional challenge due to their high potential for transmission/infection, immune evasion, and loss of vaccine efficacy as suggested in a propensity-matched analysis in morbidity and mortality of hospital-onset SARS-CoV-2 infections due to Omicron versus previous variants.^25^ A systematic review on the efficacy of a second booster dose as a strategy to mitigate the effects of Omicron variants concluded that bivalent vaccines confer greater protection by restoring lost humoral immunity and also by stimulating cellular immunity.^26^ The study suggests that the forth dose, or second booster, should be recommended for more vulnerable groups such as the elderly and immunocompromised individuals. COVID-19 vaccines have been included in the vaccination schedule of the Brazilian Ministry of Health, targeting children and priority groups, due to the risk of disease resurgence and the emergence of new variants.^27^ As this study was concluded in August 2023 and the bivalent vaccine was only made available in February of the same year, there was insufficient time to assess whether the patients adhered to bivalent vaccine. Nevertheless, an important indicator of the vaccine effectivity in the study population was the absence of symptomatic COVID-19 cases in the emergency department after the emergence of the Omicron variant.

Despite providing valuable insights into the impact of COVID-19 and vaccination on patients with SCD, this study has imitations. COVID-19 was not systematically investigated in all patients as testing was primarily performed in those presenting with respiratory symptoms or requiring hospitalization. This may have led to an underestimation of asymptomatic or mild SARS-CoV-2 infections and their potential role as a trigger for VOCr. The lack of a systematic evaluation of adverse vaccine effects limits conclusions about vaccine safety in this cohort. Additionally, the small number of COVID-19-positive cases and the absence of severe outcomes or deaths directly attributable to COVID-19 make it challenging to establish definitive associations. Future studies with systematic SARS-CoV-2 testing, larger sample sizes, and prospective monitoring of clinical outcomes and immune responses are warranted to confirm these findings and further explore the relationship between COVID-19, vaccination and VOCr.

## Conclusion

Contrary to initial expectations, the impact of the COVID-19 pandemic on patients with SCD was less severe than anticipated. Emergency department visits during the pandemic occurred primarily due to pain crises and SARS-CoV-2 infection did not appear to be a significant trigger for VOCr, severe COVID-19 outcomes, or deaths in this population. Instead, the virus may have acted as an incidental and often asymptomatic co-infection. High adherence to COVID-19 vaccination, comparable to that observed in the general population, likely played a key protective role. Interestingly, even unvaccinated individuals were not severely affected, raising questions about whether social isolation, herd immunity, or unidentified biological factors contributed to this. Further studies are needed to explore these possibilities. With COVID-19 vaccines now included in Brazil's 2024 vaccination schedule, it is crucial to address vaccine hesitancy among SCD patients and promote continued vaccination adherence as COVID-19 remains a vaccine-preventable disease.

## Conflicts of interest

The authors declare no conflicts of interest.
